# Levelling up health: A practical, evidence-based framework for reducing health inequalities

**DOI:** 10.1016/j.puhip.2022.100322

**Published:** 2022-09-22

**Authors:** Fiona Davey, Vic McGowan, Jack Birch, Isla Kuhn, Anwesha Lahiri, Anna Gkiouleka, Ananya Arora, Sarah Sowden, Clare Bambra, John Ford

**Affiliations:** a Cambridge Public Health, University of Cambridge, United Kingdom; b NIHR School for Public Health Research, United Kingdom; c NIHR Applied Research Collaboration East of England, United Kingdom; d Population Health Sciences Institute, Faculty of Medical Sciences, Newcastle University, United Kingdom; e NIHR Applied Research Collaboration North East and North Cumbria, United Kingdom

**Keywords:** Levelling up, Health inequalities, Evidence-based framework

## Abstract

There are substantial inequalities in health across society which have been exacerbated by the COVID-19 pandemic. The UK government have committed to a programme of levelling-up to address geographical inequalities. Here we undertake rapid review of the evidence base on interventions to reduce such health inequalities and developed a practical, evidence-based framework to ‘level up’ health across the country.

This paper overviews a rapid review undertaken to develop a framework of guiding principles to guide policy. To that end and based on an initial theory, we searched one electrotonic database (MEDLINE) from 2007 to July 2021 to identify published umbrella reviews and undertook an internet search to identify relevant systematic reviews, primary studies, and grey literature. Titles and abstracts were screened according to the eligibility criteria. Key themes were extracted from the included studies and synthesised into an overarching framework of guiding principles in consultation with an expert panel. Included studies were cross checked with the initial theoretical domains and further searching undertaken to fill any gaps.

We identified 16 published umbrella reviews (covering 667 individual studies), 19 grey literature publications, and 15 key systematic reviews or primary studies. Based on these studies, we develop a framework applicable at national, regional and local level which consisted of five principles - 1) **healthy-by-default and easy to use initiatives**; 2) **long-term, multi-sector action**; 3) **locally designed focus**; 4) **targeting disadvantaged communities**; and 5) **matching of resources to need.**

Decision-makers working on policies to level up health should be guided by these five principles.

## Graphical abstract

Graphical Abstract: A practical, evidence-based framework to levelling up health.

**Figure:**
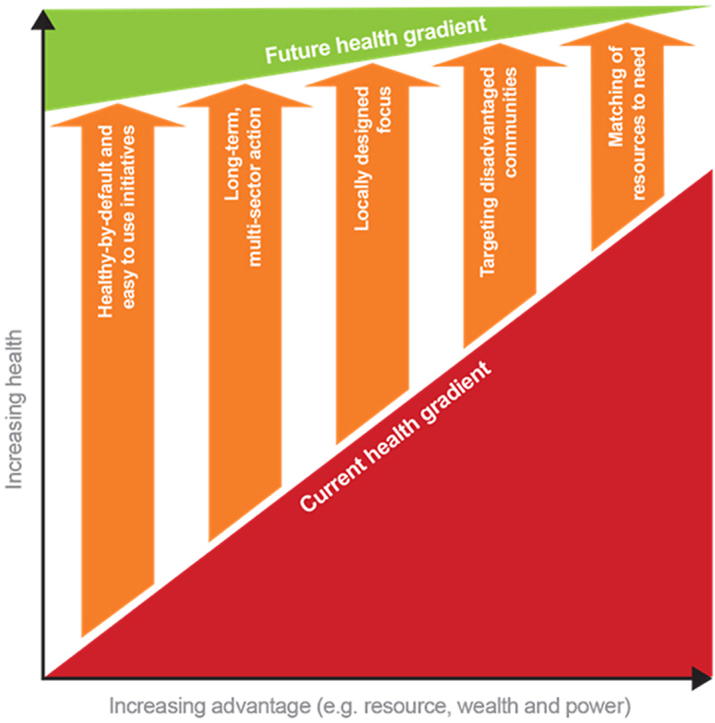

## 1 Introduction

Health inequalities - the systematic differences in health between social groups, places, or across the socio-economic gradient - exist both within and across all countries [1]. Since 2020, we have witnessed a rapid compounding of these existing health inequalities due to the COVID-19 pandemic. Unequal outcomes are being documented across the globe, particularly for disadvantaged and marginalised groups such as those with low socioeconomic status, migrant or minority ethnic groups [[2], [3], [4]]. In England, deaths in the most deprived areas of the country are double those in the least deprived and up to three times higher in minority ethnic groups [2,5]. The true impact on inequalities is expected to be much greater due to the long-term economic repercussions of the pandemic including increased unemployment, food and housing insecurity, debt, and poverty [6], which are likely to disproportionally affect people living in areas of higher deprivation and minority ethnic groups [7].

Governments around the world are seeking to address societal inequalities. Before the pandemic, the UK Government committed to a programme of ‘Levelling Up’ to help left behind areas and regions to recover and prosper to the same extent as other parts of the country. The programme, galvanised by the inequalities from the pandemic, includes investing £830million to transform high streets in 57 local areas, £10million to support improvement for local authorities with lower educational outcomes, £18million to expand the opportunities areas programme to help vulnerable and disadvantaged young people into work, and moving 22 000 civil services roles outside London and the South East [8]. To support the programme, a new No.10/Cabinet Office Level Up Unit was established and a Levelling Up White Paper [9] was published in February 2022. Health has always been a key part of the levelling up agenda, but details have not yet been forthcoming.

While there is a strong literature on reducing health inequalities [[10], [11], [12], [13]], none is framed from a ‘levelling up’ health approach. Key evidence-based principles are urgently needed to inform the levelling up for health programme. Therefore, this study set out to conduct a policy-focused rapid review of the research literature to develop a practical, evidence-based framework to level up health by area which can be implemented by a diversity of actors (e.g., governments or non-profits) and across a diversity of scales (e.g., local or national) and contexts (e.g., different countries).

## 2 Methods

### 2.1 Search strategy

The purpose was not to undertake a systematic review, identifying every study relating to health inequalities, but rather a rapid review to identify high-level evidence. We aimed to identify patterns in the literature to develop overarching principles to guide policy, rather than identifying a list of discrete interventions. We sought principles which would be true in most contexts and at different levels (e.g national, regional and local), acknowledging that these would be patterns in the literature rather than rules. To navigate the breadth of inequalities literature, we developed an initial theory of factors that influence geographical health inequalities (detailed in Appendix 1) developed by the research team, in consultation with an expert and a public panel and based on existing research [14]. The expert panel consisted of six people representing local authorities, think tanks, royal colleagues, and academia. There were two meetings of the expert panel in addition to commenting on the initial project outline and final report. To further contain the scope, we focused primarily, but not exclusively, on umbrella reviews (i.e. reviews of reviews), in additional to grey literature.

In collaboration with an experienced information scientist and librarian (IK) and based on the initial theory, we searched one electronic database (MEDLINE) from 2007 to July 2021 using the search strategy detailed in Appendix 2 to identify all the published health inequalities umbrella reviews. One researcher (AL) screened titles and abstracts according to an inclusion/exclusion criteria and a second researcher (JB) checked them; disagreements were resolved through discussion with a third author (JF).

#### 2.1.1 Inclusion criteria

• Umbrella reviews
• Interventions with a place-based approach to levelling up or aiming to reduce geographical health inequalities
• Studies based in high income countries as defined by the World Bank
• Studies with a comprehensive search strategy and quality assessment process
• Studies published in English
• Studies with any health-related outcome (e.g., morbidity, mortality, heath care access, health related practices)

#### 2.1.2 Exclusion criteria

• Studies published before 2007 covered by our previous review [13].
• Conference abstracts, commentaries, opinion pieces, editorials
• Studies not examining health inequalities by socio-economic status, geography, or area measures
• Scoping or mapping reviews or reviews only of associations (i.e., those which do not describe interventions)
• Studies which have been superseded by a more up to date review

We undertook a broad grey literature search using the key words health inequalities and levelling up health, in an internet search engine (Google) and targeted websites (Kings Fund, Health Foundation, Institute of Health Equity). Grey literature documents were reviewed to identify those which address interventions to reduce socio-economic inequalities or actions to support levelling up. To identify any further key literature, we conducted a snowball search: 1) a review of the references and sources used in these documents; and 2) citation follow-up of reviews included from the broader search above. The grey literature search identified key reviews and primary studies which were included (e.g. evaluation of the previous English health inequalities strategy). Included studies were compared to the initial theory and further targeted searching undertaken to fill any gaps (e.g. welfare).

Data extraction was carried out by one researcher (AL) and checked for accuracy by a second researcher (JB). Data was extracted regarding the aim, domains covered, and key findings for published studies. Data were then mapped against the initial theory of geographic inequalities. Next, two researchers (FD and JF) synthesised the literature via an inductive process to identify themes related to effective reductions in health inequalities. Theme headings were brought together to create a framework of guiding principles highlighting how actions to level up might reduce health inequalities. The framework was iteratively refined by the wider research team and expert panel. Due to time constraints, no formal quality assessment was undertaken.

## 3 Results

**Fig. 1:**
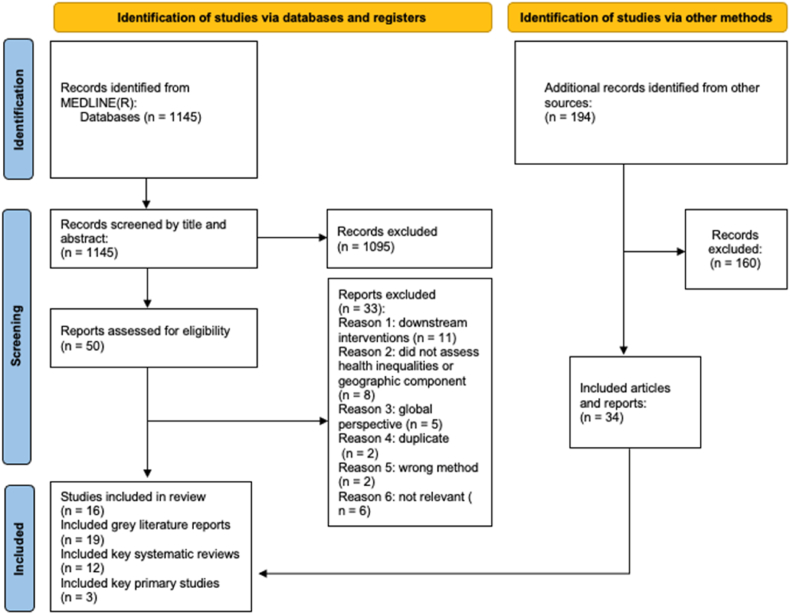
PRISMA diagram.

We screened titles and abstracts of 1145 studies and included 16 published umbrella reviews [[15], [16], [17], [18], [19], [20], [21], [22], [23], [24], [25], [26], [27], [28], [29], [30]]. Nineteen grey literature reports [[31], [32], [33], [34], [35], [36], [37], [38], [39], [40], [41], [42], [43], [44], [45], [46], [47], [48], [49]], 12 systematic reviews [[50], [51], [52], [53], [54], [55], [56], [57], [58], [59], [60], [61]], and 3 primary studies [[62], [63], [64]] were also included (see Fig. 1). Included umbrella reviews were published between 2011 and 2020 and covered a total of 667 reviews or studies (the number of each is undifferentiated as some umbrella reviews reported the number of primary studies covered by included systematic reviews, but not all). Studies covered interventions related to housing, traffic, food systems, childhood obesity, parenting, physical activity, the built/natural environment, alcohol use, and adolescent health. Several reviews also examined impacts on health inequalities by types of intervention delivery and macroeconomic conditions. An overview of study characteristics for the included articles is shown in Table 1.

**Table 1:** Study characteristics of included literature.

First author/publisher (year)	Title	Publication/Study Type	Aim	No. of included systematic reviews or studies	Domains covered (see Appendix 1)	Regional Context
Umbrella reviews						
**Gibson (2011)** [15] (International)	Housing and health inequalities: A synthesis of systematic reviews of interventions aimed at different pathways linking housing and health	Umbrella review	Identify what types of housing and neighbourhood interventions have been reviewed systematically and how these relate to the different pathways between housing and health; establish what gaps exist in the systematic review evidence base on housing interventions; and consider what existing reviews can tell us about the impact of housing and neighbourhood interventions on health and health inequalities	5 SRs	Social: housing quality/cost	USA, UK, New Zealand, Europe
**Cairns (2015)** [16] (International)	Go slow: an umbrella review of the effects of 20 mph zones and limits on health and health inequalities	Umbrella review	Examine the effects of 20mph zones and limits on health and health inequalities	5 SRs	Local physical: infrastructure & transport	UK, USA, Europe
**Welch (2016)** [23] (International)	Interactive social media interventions to promote health equity: an overview of reviews	Umbrella review	Assess the effects of interactive social media interventions on health outcomes, behaviour change and health equity	11 SRs	Health behaviours	USA, UK, Australia, Canada, Europe
**Haby (2016)** [24] (International)	Agriculture, food, and nutrition interventions that facilitate sustainable food production and impact health: An overview of systematic reviews	Umbrella review	Identify the agriculture, food, and nutrition security interventions that facilitate sustainable food production and have a positive impact on health	15 SRs	Social and local physical	Developing, mostly developed, and developed countries
**Cauchi (2016)** [25] (International)	Environmental components of childhood obesity prevention interventions: an overview of systematic reviews	Umbrella review	Summarise the evidence reported in systematic reviews on the effectiveness of population-level childhood obesity prevention interventions that have an environmental component	63 SRs	Local physical, health behaviours	Not reported
**Anderson (2018)** [26] (International)	City-based action to reduce harmful alcohol use: review of reviews	Umbrella review	Investigate the potential impact of city-based action to reduce the harmful use of alcohol amongst adults	5 SRs	Health behaviours: drugs and alcohol	North America, Nordic countries, Australia, New Zealand
**Bird (2018)** [27] (International)	Built and natural environment planning principles for promoting health: an umbrella review	Umbrella review	Assess relationships between the built and natural environment and health, concentrating on five topic areas: neighbourhood design, housing, food environment, natural and sustainable environment, and transport	117 SRs	Local environment: built environment, housing, infrastructure, green space; social: housing quality/cost	High- and middle- income countries (Europe, North America, Australasia, and Japan)
**Craike (2018)** [28] (International)	Interventions to improve physical activity among socioeconomically disadvantaged groups: an umbrella review	Umbrella review	Examine the effectiveness of interventions to improve physical activity among socioeconomically disadvantaged groups; the characteristics of effective interventions; and directions for future research	17 SRs	Physical activity	Not reported
***Pierron* (2018)** [29] (International)	Supporting parenting to address social inequalities in health: a synthesis of systematic reviews	Umbrella review	Analyse components and characteristics of effective interventions in parenting support and the extent to which the reviews considered social inequalities in health	21 SRs	Social	USA, UK, Europe
**Thomson (2018)** [17] (International)	The effects of public health policies on health inequalities in high-income countries: an umbrella review	Umbrella review	Examine the effects of public health policies on health inequalities in high-income welfare states	29 SRs	Policy and politics	Majority USA, EU-28 members, high income countries
**Thomson (2019)** [30] (International)	The effects of community pharmacy-delivered public health interventions on population health and health inequalities: A review of reviews	Umbrella review	Assess the effectiveness of community pharmacy-delivered public health services and assess how they impact on inequalities in health using PROGRESS-Plus characteristics	15 SRs	Policy and politics: healthcare system	UK, USA & Puerto Rico, Europe, Australia, Canada, Japan, Korea, South Africa, Thailand
**Naik (2019)** [18] (International)	Going upstream – an umbrella review of the macroeconomic determinants of health and health inequalities	Umbrella review	Identify the evidence for the health and health inequalities impact of population-level macroeconomic factors, strategies, policies and interventions	62 SRs	Economic, policy and politics	Majority high and middle-income countries
**McCartney (2019)** [19] (International)	Impact of political economy on population health: a systematic review of reviews	Umbrella review	Understand the extent to which political economy, and important aspects of it, explain differences in health outcomes within and between populations over time	58 SRs	Policy and politics	Europe, USA, UK, southeast Asia, Canada, Mexico, sub-Saharan Africa, Bangladesh, Peru, Madagascar,
**Carey (2019)** [20] (International)	Personalisation schemes in social care and inequality: review of the evidence and early theorising	Umbrella review	Conduct a systematic review of the evidence of personalisation schemes and their likely effects on inequality	6 SRs	Policy and politics: health care system	Not reported
**Macintyre (2020)** [21] (International)	Socioeconomic inequalities and the equity impact of population-level interventions for adolescent health: an overview of systematic reviews	Umbrella review	Examine systematic review evidence on the equity impact of population-level interventions intended to improve health, happiness and wellbeing for adolescents	140 SRs	Health behaviours	Not reported (relevance to UK/Scotland was an inclusion criteria)
**Garzón-Orjuela (2020)** [22] (International)	An overview of reviews on strategies to reduce health inequalities	Umbrella review	Identify and synthesize strategies or interventions that facilitate the reduction of health inequalities	98 SRs	Economic, social, and policy and politics	Not reported
Grey literature reports						
**Public Health Research Consortium (2008)** [31] (International)	Tackling the wider social determinants of health and health inequalities: evidence from systematic reviews	Report	Identify existing systematic reviews and relevant primary studies, and to use these to identify priorities for new systematic reviews and for new primary studies of interventions addressing inequalities in health.	32 SRs 16 studies	Economic, social, and policy and politics	Developed/OECD countries
**British Academy for the humanities and social sciences (2014**) [32] (National)	"If you could do one thing … ". Nine local actions to reduce health inequalities	Report	Identify where, and how, the social sciences can contribute to reducing health inequalities	N/A	Policy and politics	UK
**The Scottish Government (2015)** [42] (National)	Tackling Inequalities in the Early Years: Key messages from 10 years of the Growing up in Scotland study	Report	Highlight how the study has contributed to the evidence base on children and families in Scotland, on the extent of and how to reduce inequalities in outcomes in the early years.	N/A	Economic, social, policy and politics	Scotland
**Public Health England and Institute of Health Equity (2015)**^43^(National)	Using the Social Value Act to reduce health inequalities in England through action on the social determinants of health	Report	Explain what social value means, and how and whether it is used	N/A	Policy and politics	England
			Set out the reasons to act on social value			
			Provide information, guidance and examples of local action for local public sector commissioners in order to increase social value in their procurement activities			
**NHS Health Scotland (2015)** [44] (National)	Health inequalities: What are they? How do we reduce them?	Report	Promote action to reduce health inequalities	N/A	Policy and politics	Scotland
**Public Health England (2018)** [46] (National)	Which service or policy mechanisms, models or approaches, have been shown to be effective or ineffective at reducing the inequalities that are known to have an impact on childhood obesity?	Report	A briefing document that aimed to summarise best available evidence on the approaches and interventions that may reduce the inequalities that impact on obesity in childhood.	N/A	Policy and politics, health behaviours	England
**Public Health England (2018)** [47] (National)	Which service or policy mechanisms, models or approaches, have been shown to be effective or ineffective at reducing inequalities in access to health and social care services?	Report	A briefing document that aimed to summarise best available evidence on the interventions, models and approaches to reduce inequalities in access to health and social care services.	N/A	Policy and politics	England
**Public Health England (2018)** [48] (National)	Which service or policy mechanisms, models or approaches, have been shown to be effective or ineffective at reducing the inequalities that older people experience?	Report	A briefing document that aimed to summarise best available evidence on service delivery mechanisms, models or approaches that have been shown to be effective or ineffective at reducing the inequalities that older people experience.	N/A	Policy and politics	England
**Public Health England (2018)** [49] (National)	Which service or policy mechanisms, models or approaches, have been shown to be effective at reducing educational inequalities in early years?	Report	A briefing document that aimed to summarise best available evidence on service delivery mechanisms, models or approaches that have been shown to be effective at reducing educational inequalities in early years.	N/A	Policy and politics	England
**Public Health England (2018)** [33] (National)	Which service or policy mechanisms, models or approaches, have been shown to be effective or ineffective at reducing inequalities in employment?	Report	A briefing document that aimed to summarise best available evidence on interventions that have been effective, or ineffective, at reducing equalities in employment.	N/A	Economic: labour market; policy and politics	England
**Local Government Association (2020)** [34] (National)	Social determinants of health and the role of local government	Report	Identify what local government can do to improve health by tackling social determinants of health	N/A	Policy and politics	England
**Local Government Association (2020)** [35] (National)	Public health transformation seven years on. Prevention in neighbourhood, place and system	Report	Local Government Association 2020 public health annual report	N/A	Policy and politics	England
**Public Health Wales (2020)** [36] (National)	Digital technology and health inequalities: a scoping review	Report	Understand and offer advice on how equality can be promoted or risks mitigated in the design and use of digital technologies.	84	Policy and politics	Wales
			Inform a theoretical framework for considering how lack of access, skills and motivation for using digital technologies (digital exclusion) could affect health outcomes.			
**The Health Foundation (2020)** [37] (National)	Using economic development to improve health and reduce health inequalities	Report	Provide a framework for practitioners to consider the interventions available and implement strategies most appropriate to their local situation.	N/A	Economic, policy and politics	UK
**Institute of Health Equity (2020)** [38] (National)	Health Equity in England: The Marmot Review 10 Years On	Report	Explore what has happened to health inequalities and social determinants of health in the decade since the Marmot	N/A	Economic, social, policy and politics	England
			Review. Provide in-depth analysis of health inequalities in England and assess what has happened in key social determinants of health, positively and negatively, in the last 10 years.			
			Set out an agenda for the Government and local authorities to take action to reduce health inequalities in England.			
**Institute of Health Equity (2020)** [39] (National)	Coventry – A Marmot City. An evaluation of a city-wide approach to reducing health inequalities	Report	Understand the strategic impact of the Marmot City approach in Coventry and the impact on population outcomes.	N/A	Economic, social, policy and politics	England
			Inform future developments in Coventry.			
			Provide information and insight for other areas who are developing system wide and integrated approaches to reducing health inequalities.			
			Provide evidence and analysis for a broad range of stakeholders in UK and globally including for the Marmot Ten Years on work.			
**Institute of Health Equity (2021)** [40] (National)	Build Back Fairer in Greater Manchester: Health Equity and Dignified Lives	Report	Provide evidence of the health inequality challenges the Greater Manchester City Region will face post-pandemic and to make recommendations to monitor and reduce them.	N/A	Economic, social, policy and politics	England
**Institute for Public Policy Research North (2021)** [41] (National)	Women in the North. Choosing to challenge inequalities.	Report	Challenge thinking to fully understand how different inequalities interact with one another.	N/A	Economic, social, policy and politics	England
**Public Health England (2021)** [73] (National)	Place-based approaches for reducing health inequalities: main report	Report	Create a place-based approach to support local areas in addressing health inequalities by identifying strategic and system-wide action has previously reduced population-wide health inequalities.	N/A	Economic, social, local environment, policy and politics	England
Key systematic reviews and primary studies						
**Eyles (2020)** [50] (International)	Food pricing strategies, population diets, and non-communicable disease: a systematic review of simulation studies	Systematic review	Review simulation studies investigating the estimated association between food pricing strategies and changes in food purchases or intakes (consumption) (objective 1); Health and disease outcomes (objective 2), and whether there are any differences in these outcomes by socio-economic group (objective 3).	32	Economic; Social: food availability; Health behaviours: diet	OECD countries
**Brown (2014)** [51] (International)	Equity impact of interventions and policies to reduce smoking in youth: systematic review	Systematic review	Assess the impact of individual-level smoking cessation interventions undertaken in Europe since 1995, on socioeconomic inequalities in adult smoking	38	Health behaviours: smoking	USA, UK, Germany, New Zealand, Australia, Canada, Finland, France, Israel, The Netherlands, Portugal, Spain, Sweden
**Barr (2014)** [62] (National)	The impact of NHS resource allocation policy on health inequalities in England 2001–11: longitudinal ecological study	Longitudinal ecological study	Investigate whether the policy of increasing National Health Service funding to a greater extent in deprived areas in England compared with more affluent areas led to a reduction in geographical inequalities in mortality amenable to healthcare.	N/A	Policy and politics: health care system	England
**Beauchamp (2014)** [54] (International)	The effect of obesity prevention interventions according to socioeconomic position: a systematic review	Systematic review	Identify interventions for obesity Systematically review the effectiveness of worprevention that evaluated a change in adiposity according to socioeconomic position (SEP) and to determine the effectiveness of these interventions across different socioeconomic groups.	14	Health behaviours: diet and physical activity	USA, The Netherlands, France, Germany, Australia
**Durand (2014)** [55] (International)	Do interventions designed to support shared decision-making reduce health inequalities? A systematic review and meta-analysis	Systematic review and meta-analysis	Evaluate the impact of shared decision-making (SDM) interventions on disadvantaged groups and health inequalities.	19	Policy and politics: health care system	USA, Australia, Nicaragua
**Cairns (2014)** [56] (International)	Weighing up the evidence: a systematic review of the effectiveness of workplace interventions to tackle socio-economic inequalities in obesity	Systematic review	Systematically review the effectiveness of workplace interventions in reducing socio-economic inequalities in obesity	18	Health behaviours: diet and physical activity	USA, Chile, Brazil, Australia, South Korea, Germany
			Establish how such interventions are organized, implemented and delivered.			
**Hillier-Brown (2014)** [58] (International)	A systematic review of the effectiveness of individual, community and societal level interventions at reducing socioeconomic inequalities in obesity amongst children	Systematic review	Systematically review studies of the effectiveness of interventions (individual, community and societal)	23	Health behaviours: diet and physical activity	USA, Australia, Brazil, Chile, Peru, Israel, the Netherlands, Finland, France, Switzerland
			operating via different approaches (targeted or universal) in reducing socio-economic inequalities in obesity-related			
			outcomes amongst children.			
**Brown (2014)** [57] (International)	Equity impact of European individual-level smoking cessation interventions to reduce smoking in adults: a systematic review	Systematic review	Assess the equity impact of interventions/policies on smoking.	29	Health behaviours: smoking	Europe
**Harris (2015)** [59] (International)	Can community-based peer support promote health literacy and reduce inequalities? A realist review	Realist review	Develop a better understanding of the potential for community-based peer support (CBPS) to promote better health literacy (HL).	570	Social	USA, UK
**McGill (2015)** [60] (International)	Are interventions to promote healthy eating equally effective for all? Systematic review of socioeconomic inequalities in impact	Systematic review	Review of interventions to promote healthy eating to identify whether impacts differ by socioeconomic position (SEP).	36	Health behaviours: diet	Europe, North America, Australia, New Zealand, UK
**Moore (2015)** [61] (International)	Socioeconomic gradients in the effects of universal school-based health behaviour interventions: a systematic review of intervention studies	Systematic review	Report a content analysis of discussion of socioeconomic inequality within the rationale for interventions and interpretation of findings within published articles of school-based interventions.	98	Health behaviours: diet and physical activity	Europe, North America, Australasia, South America, Asia
**Crocker-Buque (2016)** [52] (International)	Interventions to reduce inequalities in vaccine uptake in children and adolescents aged <19 years: a systematic review	Systematic review	Update a 2009 systematic review on effective interventions to decrease vaccine uptake inequalities considering new technologies applied to vaccination and new vaccine programmes (e.g., human papillomavirus in adolescents).	41	Policy and politics: Health care system	USA, UK, Canada, Australia
**Barr (2017)** [63] (National)	Investigating the impact of the English health inequalities strategy: time trend analysis	Time trend analysis	Investigate whether the English health inequalities strategy was associated with a decline in geographical health inequalities, compared with trends before and after the strategy.	N/A	Policy and politics: Public health regulation	England
**Griffin (2019)** [64] (National)	Evaluation of intervention impact on health inequality for resource allocation	Economic evaluation	Demonstrate a method for conducting quantitative inequality impact assessment using available aggregate data.	N/A	Policy and politics: social policies	England
**Simpson (2021)** [53] (International)	Effects of social security policy reforms on mental health and inequalities: A systematic review of observational studies in high-income countries	Systematic review	Provide a synthesis of observational literature on the effects on mental health and inequalities in mental health of social security reforms.	21	Wider economic: Welfare system	USA, UK, Canada, South Korea, Chile, Germany, Australia, the Netherlands

### 3.1 A practical, evidence-based framework to levelling up health

**Fig. 2:**
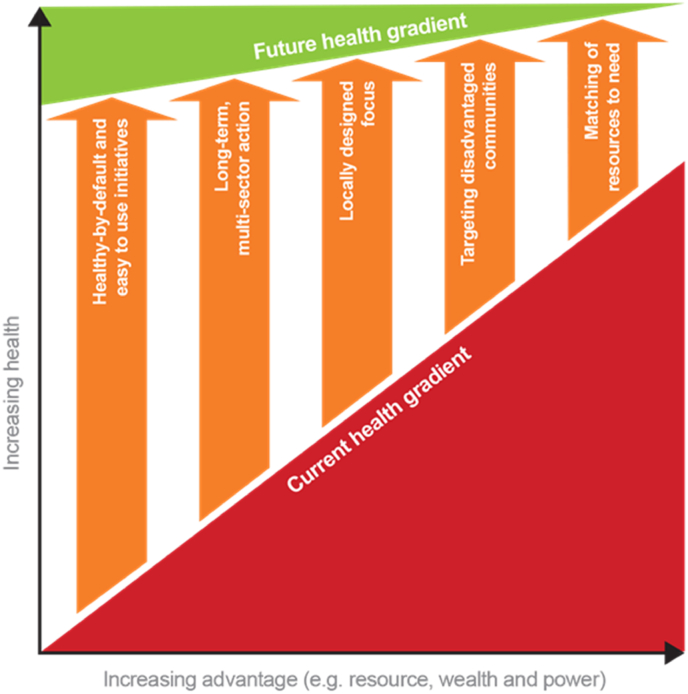
A practical, evidence-based framework to levelling up health.

Five key themes were identified and combined into an evidence-based framework of principles which highlights the need to flatten the health gradient (i.e., level up) while simultaneously improving the health of all (see Fig. 2). The five principles are 1) **healthy-by-default and easy to use initiatives**; 2) **long-term, multi-sector action**; 3) **locally designed focus**; 4) **targeting disadvantaged communities**; and 5) **matching of resources to need.** All the principles are supported by a robust evidence base (see Table 2) and are applicable at a national, regional, and local level. They are overlapping, rather than mutually exclusive, and should be implemented in conjunction with each other.

**Table 2:** Summary of the published evidence contributing to each principle.

Principle	First author (year)	No. of included reviews	Domains covered	Evidence
**Healthy-by-default and easy to use initiatives**	Thomson (2018) [17]	29 SRs (150 studies)	Tobacco, alcohol, nutrition, reproductive health, infectious disease control, the environment, workplace regulations	• 2 studies found USA food stamp (subsidy) programme had positive impacts on foetal survival and weight gain during pregnancy of low-income populations. • 9 studies 10–20% increased intake of targeted foods or nutrients of participants in food subsidy programme • 4 studies of taxes on unhealthy foods and drink showed positive equity effects on diet outcomes • 1 SR found significant drop in casualties in the more deprived areas, compared to the less deprived areas from speed limit interventions. • 1 study found reduced absolute inequalities in dental caries between the most affluent and least affluent areas associated with intervention that provided fluoridated toothpaste and daily toothbrushing supervision for 5-year-olds. • 2 studies found evidence that fiscal incentive schemes (maternity allowance, childcare benefits) may decrease inequalities in vaccination rates.
	Eyles (2012) [50]	32 studies	Nutrition and diet	• 11 out of 14 studies reporting impacts by SES found pro-health and pro-equity outcomes for food taxes and subsidies (although many note that taxes would be regressive with more financial burden on low-income individuals).
	McGill (2015) [60]	36 studies	Nutrition and diet	• 10 of 18 “price” interventions were likely to reduce inequalities by improving healthy eating outcomes more for individuals of low SES, particularly when interventions were a combination of taxes and subsidies with all 6 respective studies reducing inequalities. • 4 of 6 “place” interventions reduced inequalities and none widened them. • 8 of 19 “person” (individual-based information and education) interventions widened inequalities.
	Cauchi (2016) [25]	63 SRs	Childhood obesity	• 48 studies with positive outcomes reported the following effective environmental strategies: improving overall school food environment (nutrition standards, reformulating school lunches, removing vending machines/banning sale of sugar sweetened beverages/snacks high in fat, sugar, or salt), purchasing new PE/sports equipment, daily formal physical activity sessions, providing free or low-cost fruit, making playgrounds available for physical activity after school hours, providing free/low-cost water, providing healthy breakfasts at school, substituting sweetened beverages, reducing screen time at home.
	Beauchamp (2014) [54]	14 studies	Obesity	• 5 of 6 interventions with a positive equity impact included structural changes to support behaviour change, 5 had a wide reach (3 community-based and 2 school-based), and all were multi-year in duration. • 4 of 5 interventions with no beneficial impact among lower SES groups had low structural changes and 1 had moderate amounts of structural change, 3 were very short term (2–10 weeks), and 4 were based solely on information delivery.
	Durand (2014) [55]	19 studies	Shared decision-making	• 5 of 7 studies differentiating outcome by disadvantage/literacy levels reduced disparities in knowledge, decisional conflict, uncertainty and treatment preferences suggesting SDM interventions could narrow health disparities by promoting skills/resources needed to engage in SDM.
	Moore (2015) [61]	20 studies	Universal school-based interventions on health behaviours	• Of 4 education-based interventions, 1 widened inequalities and 3 had a neutral effect. • Of 4 environmental interventions, 1 reduced inequalities and 3 had a neutral effect. • Interventions combining education and environmental change had mixed results.
	Carey (2019) [20]	6 studies	Personalisation schemes	• Accessing and benefiting from schemes based on personalisation requires high levels of skills and resources at the individual level. • Identified factors associated with better outcomes in personalisation schemes were higher levels of economic, cultural, social, and symbolic capital in the forms of education, being employed, having capable networks and support, knowledge and skills in navigating complex systems, household income, knowledge of where to access information and the capacity to self-manage individual budgets.
	Cairns (2015) [56]	18 studies	Obesity	• 0 of 11 counselling or advice-based interventions reduced inequalities in obesity.
	Craike (2018) [28]	17 SRs	Physical activity	• 1 SR found that 2 of 4 universal policies showed a positive equity impact on children's physical activity levels: provincial school physical education policy requiring students to take physical education to graduate from secondary school and a children's fitness tax credit.
	Haby (2016) [24]	15 SRs, 7 economic evaluations	Agriculture, food, nutrition	• 1 SR reported on health inequality impact found reduction in health inequalities from balancing taxes on unhealthy foods with subsidies on healthy food.
**Long-term, multi-sector action**	Gibson (2011) [15]	5 SRs (130 studies)	Housing and neighbourhood conditions	• 1 SR (72 studies) found highest efficacy in interventions aimed at multiple pathways (rehousing and changes to: indoor equipment or furniture; respondents' knowledge or behaviour; community norms or collective behaviour; housing policy or regulatory practices, and health practitioners' behaviour) and which are ecological (target multiple levels (i.e. individuals, households, housing and neighbourhoods)).
	Craike (2018) [28]	17 SRs	Physical activity	• 3 reviews on children found that physical activity interventions, particularly those that were school-based and multicomponent were likely to be effective. Common elements of successful policy-focused interventions included enhancements to physical education, additional physical activity opportunities, school self-assessments, and education about physical activity. • 1 SR on all age groups found intensive interventions are most likely to reduce socio economic status inequalities in physical activity.
	McGill (2015) [60]	36 studies	Nutrition and diet	• 4 out 6 place-based interventions demonstrated to reduce inequalities were implemented in a range of settings including schools, workplaces, and communities/neighbourhoods.
	Naik (2019) [18]	62 (umbrella, meta-analyses, & narrative)	Macroeconomic determinants	• High quality SR showed evidence of pro-equity impact from taxing tobacco and moderate quality SR found mixed, but mostly positive impact on reductions in preterm births among mothers with low education and black mothers. Supported by findings of 4 other lower quality reviews. • 3 reviews (low quality) found some association between unemployment insurance and reduced inequalities and better health outcomes. • 4 reviews (moderate to low quality) on gendered health inequalities found positive equity impacts from the dual-earner policy model and welfare conditions reducing job precarity. • 2 reviews (moderate quality) found pro-equity impacts of occupational health and safety regulations such as preventing toxin exposures.
	Simpson (2021) [53]	38 studies	Social security policy and mental health	• 14 of 21 studies on expansionary policies (increased benefit amount or access) improved mental health; 4 studies evaluated inequalities of which 2 reduced inequalities and 2 had no impact. • 11 of 17 studies on contractionary policies (decreased benefit amount or access) worsened mental health; 10 evaluated inequalities which widened in 3, narrowed in 2, and had mixed or no effects in 5.
	Macintyre (2020) [21]	15 SRs (1720 studies)	Adolescent health	• Evidence for market regulation impact in SR on youth smoking found 7 (of 38) studies showed positive impact on inequalities, 16 showed neutral effects, 12 negative impact, 4 mixed and 1 unclear. Taxation/increasing the price of cigarettes had the most evidence for positive equity impact.
**Locally designed focus**	Cauchi (2016) [25]	63 SRs	Childhood obesity	• Environmental interventions had beneficial equity impacts (ES: 0.09 [0.16, 0.02]). • Community-based interventions of any type & parental involvement resulted in small but consistently positive ES ranging from 0.094 [p = <0.001] to 0.151 [0.334, 0.031].
	Craike (2018) [28]	17 SRs	Physical activity	• 1 SR on interventions with pre-schoolers: 6 of 11 included studies showed a significant effect; all 3 community-based interventions were effective. • 9 SRs on adults found factors associated with higher effectiveness were: the involvement of the community in the design and implementation of interventions; developing community infrastructure to sustain effective interventions; interventions delivered through personal contact; and tailored interventions. • 1 SR on all age groups found community settings were the most effective intervention setting for socioeconomically disadvantaged groups.
	Crocker-Buque (2016) [52]	41 studies	Immunisation	• 16 studies on multicomponent locally designed interventions demonstrated higher efficacy from improving immunisation in children and adolescents in the short term for ethnically diverse, low-income populations.
	Pierron (2018) [29]	21 SRs	Supporting parenting	• 1 SR found increased effectiveness from diversifying approaches shared between state, school, and neighbourhood organisations and varying intervention to local context and different cultures/societies. • 2 SRs reported on necessity of integrating the entire network related to parenting (environment, professionals, organisations, social contexts, etc.).
	Thomson (2019) [30]	15 SRs (157 studies)	Community pharmacy-delivered interventions	• 17 studies found increased vaccination rates among people who had missed vaccination the previous year or otherwise wouldn't have accessed vaccination services with pharmacy-delivery and that of those delivered a third of the vaccinations took place outside traditional working hours documenting the increased accessibility provided by community pharmacy networks. • 1 study found increased breast and cervical cancer screening uptake among low- and moderate-income women.
**Targeting disadvantaged communities**	Moore (2015) [61]	20 studies	Universal school-based interventions on childhood health behaviours	• 10 of 20 universal interventions had a neutral impact on inequalities. • 6 of 20 universal interventions widened inequalities.
	Thomson (2018) [17]	29 SRs (150 studies)	tobacco, alcohol, nutrition, reproductive health, infectious disease control, the environment, workplace regulations	• 3 studies documented a widening of socio-economic inequalities from mass media intervention for pre-conception folic acid use from the national campaign (which persisted for 3 years), but not in the local campaign. The studies showed worsening health inequality effects in terms of folate uptake by education level, and the prevalence of neural tube defects by ethnicity. • 1 SR found that the Expanded Food and Nutrition Education Program (EFNEP) – a federal community outreach programme targeted at low-income families – increased fruit and vegetable consumption and had a positive effect on health inequalities. • 2 studies found interventions targeted toward disadvantaged groups increased screening rates – particularly amongst lower socio-economic groups. • 4 studies found positive effects of ‘reminder and recall’ systems when targeted at disadvantaged groups, but that universal systems had no effect on reducing inequalities in vaccine uptake rates. 7 studies found a combination of targeted and universal immunisations improved health outcomes for indigenous populations. • 1 study found complex interventions targeted interventions were effective in encouraging child- hood vaccination when specifically targeted at lower SES groups of younger children.
	Cairns (2015) [56]	18 SRs	Obesity	• 2 RCTs (strong/moderate quality) demonstrated reduced inequalities in physical activity interventions targeted at low-income workers. • 1 observational study (moderate quality) showed increased inequalities from a universally delivered workplace physical activity intervention.
	Bird (2018) [27]	17 SRs	Built and natural environment	• 1 SR found provision of affordable and diverse housing was found to be associated with higher or increased physical activity, primarily walking and perceived safety among those from low-income groups. • 9 SRs reported that provision of affordable housing to vulnerable individuals with specific needs (those living with intellectual disability, substance users, individuals experiencing homelessness, and those living with a chronic condition) was associated higher or improved social, behavioural, physical and mental health-related outcomes.
	Gibson (2011) [15]	5 SRs (130 studies)	Housing and neighbourhood conditions	• 30 studies found warmth and energy efficiency interventions had the clearest positive impacts on health. Interventions that reported the largest effects were targeted at vulnerable groups, including those with existing health conditions and the elderly.
	Durand (2014) [55]	19 studies	Shared decision-making	• 3 studies suggested that despite knowledge levels being lower in disadvantaged groups pre-intervention, disparities between groups tended to disappear post-intervention, particularly when the intervention was adapted to disadvantaged groups' needs (e.g. low literacy).
**Matching of resources to need**	Barr (2017) [62]		NHS resource allocation	• Between 2001 and 2011 the increase in NHS resources to deprived areas accounted for a reduction in the gap between deprived and affluent areas in male mortality amenable to healthcare of 35 deaths per 100 000 population (95% confidence interval 27 to 42) and female mortality of 16 deaths per 100 000 (10–21). This explained 85% of the total reduction of absolute inequality in mortality amenable to healthcare during this time. • Each additional £10 m of resources allocated to deprived areas was associated with a reduction in 4 deaths in males per 100 000 (3.1–4.9) and 1.8 deaths in females per 100 000 (1.1–2.4).
	Barr (2014) [63]		UK Health Inequalities Strategy	• During the strategy the gap in life expectancy for men reduced by 0.91 months each year (0.54–1.27 months) and for women by 0.50 months each year (0.15–0.86 months) compared to increasing inequalities before and after strategy implementation. • By 2012 the gap in male life expectancy was 1.2 years smaller (95% confidence interval 0.8–1.5 years smaller) and the gap in female life expectancy was 0.6 years smaller (0.3–1.0 years smaller) than it would have been if the trends in inequalities before the strategy had continued.

SR = systematic reviews.

### 3.2 Healthy-by-default and easy to use initiatives

Evidence from 11 studies (4 umbrella reviews and 7 systematic reviews) indicated the importance of healthy-by-default and easy to use initiatives which change the conditions to make health-positive choices easier. For example, changing food purchasing conditions through a combination of taxing unhealthy foods and subsidising healthy foods was consistently documented as an intervention type most likely to reduce health inequalities [17,50,60]. The efficacy of easy to engage with interventions was especially highlighted in comparison to downstream, information-giving interventions which were the most likely to widen inequalities in a variety of outcomes related to diet, weight, cholesterol levels, and folate intake [17,60]. Easy to use programmes were more likely to address inequalities, for example by providing the resources needed to engage in health promoting behaviours [17,28]; providing fluoride toothpaste for home use and daily toothbrushing supervision for 5-year-olds led to a reduction in dental health inequalties [17].

### 3.3 Long-term, multi-sector action

Long-term, multi-sector action was supported by evidence from 6 studies (4 umbrella reviews and 2 systematic reviews). In an assessment of housing and neighbourhood interventions, researchers found that a reduction in health inequalities may not have been observed for some interventions due to the reality that disadvantaged populations face many barriers [15]. An intervention aimed at one determinant alone (housing) is unlikely to be effective when individuals are still impacted by others (e.g., working conditions or access to healthy foods). Housing interventions were most likely to be effective in improving health and reducing inequalities when there were multiple interventions targeting several social determinants of health [15]. Systematic and umbrella reviews of physical activity and healthy eating interventions also show that interventions are more likely to reduce inequalities if they are more intensive, multi-component, address multiple barriers to healthy behaviours, and are based in a range of settings from schools and workplaces to churches and community centres [28,60]. Analyses of welfare states, macroeconomic conditions, and social security policies have found that different policies across all these domains are associated with health inequality [18,53].

### 3.4 Locally designed focus

An evidence base of 5 studies (4 umbrella reviews and 1 systematic review) demonstrated increased efficacy and reduced inequalities for programmes which are tailored to local contexts across various domains such as improving child immunisation rates and parenting interventions [29,52]. Including community-based infrastructure developments was associated with more sustainable physical activity interventions, maintaining increased adult physical activity levels, and reduced inequalities [28]. An umbrella review of community pharmacy-based interventions found that previously unvaccinated individuals were a third more likely to receive the influenza immunisation outside of traditional working day hours [30]. The success of peer-support programmes is also indicative of the potential for locally designed services to reduce health inequalities more effectively by adapting to the particular contexts of communities [59].

### 3.5 Targeting disadvantaged communities

There was evidence from 6 studies (4 umbrella reviews and 2 systematic reviews) that universally applied programmes which do not also target disadvantaged communities or account for their particular needs, assets, and barriers to health are less effective in reducing health inequalities and may even widen them [17,56,61]. This was observed in school-based interventions, immunisation campaigns, national media campaigns, and workplace physical activity interventions. Housing improvement interventions with the largest effects and reductions in inequalities were aimed at vulnerable and low-income groups [15,27]. Provision of benefits to disadvantaged groups may also reduce health inequalities, such as food subsidy programmes for women of low-socioeconomic status which reduced inequalities in mean birthweight and food/nutrient uptake [17].

### 3.6 Matching of resources to need

Two studies assessing the UK Health Inequalities Strategy of 1997–2010 highlighted the importance of allocating resources according to need. This type of funding formula was integrated in the English health inequalities strategy implemented between 1997 and 2010. A time trend analysis of the health inequalities strategy found an associated decline in geographically unequal life expectancies compared to increasing inequality both before and after the strategy's implementation [63]. The gap in male and female life expectancy in between the most deprived local authorities and the rest of England was smaller in 2012 by 1.2 and 0.6 years smaller, respectively, than would have been the case if trends in inequalities before strategy implementation had continued [63]. Another study found that allocation of NHS resources proportionate to geographic need – with more deprived areas receiving more resources – was associated with decreased inequalities in mortality amenable to healthcare [62]. For each £1.00 of new resources allocated to deprived areas there was a greater absolute improvement in mortality amenable to healthcare compared to each £1.00 of new resources allocated to affluent areas [62].

## 4 Discussion

### 4.1 Statement of principle findings

Here we present a practical, evidence-based framework of guiding principles to help level up health: 1) healthy-by-default and easy to use initiatives; 2) long-term, multi-sector action; 3) locally designed focus; 4) targeting disadvantaged communities; and 5) matching of resources to need. The principles are designed to collectively inform national, regional, and local policy and services.

### 4.2 What the findings mean

Progress on closing the gap is possible. The previous UK cross-government health inequalities programme reduced the socio-economic gap in life expectancy by six months *and* improved overall life expectancy – both levelling up and improving overall population health [63]. It also resulted in a reduction in the infant mortality inequalities and healthcare-amenable mortality, demonstrating that with commitment and resources meaningful change is possible [62,63]. This was only achieved through sustained, multi-component, and cross-government action over more than 10 years.

The principles described in this framework are upstream and focused on both structural changes and locally based-community engagement given the complex relationships between health outcomes, the social determinants of health, and human agency [65,66]. Until now, there has been a tendency to start with upstream factors but end up with downstream policies focused on behaviour change, such as untargeted information publicity campaigns, which may actually widen inequalities [67,68]. This so-called lifestyle drift occurs because it is easier to offer services and deliver programmes focused on providing information and warning of risks, than addressing the social structures that dictate the health of places and individuals [68]. This also likely skews the evidence base as behavioural interventions are easier to measure in short term programmes and thus are more likely to be included in the evidence base, documented as successful, and repeated. Top-down interventions which assume a one-size-fits-all approach and fail to engage with local communities are also likely to increase inequalities. Our review stresses the need to avoid these tendencies.

Health inequalities have arisen over decades, if not centuries, and have multiple different facets, but tend to have the same root cause: an unequal distribution of the wider determinants of health. There is no one initiative or programme that will address this unequal distribution of resources, opportunity, wealth, education, and power, but rather a multi-level, multi-component programme sustained over the long term is needed. In the framework, we have avoided highlighting specific domains, such as housing, welfare, employment, or education, but rather identified the guiding principles and transferrable evidence which could be applied in any government department, local authority, public health body, or NHS organisation. This is because levelling up health requires an equity-in-all approach with every sector at every level doing what they can.

### 4.3 Comparison with previous literature

The principles contained within our framework are supported by other reports. Public Health England's Place-Based Approaches to Reducing Inequalities recognises the complex causes of health inequalities and provides guidelines for different sectors to work together, implement multi-component interventions, and use of local data [69]. Our framework also aligns with the 2010 Marmot Review *Fair Society, Healthy Lives* principles which places a heavy focus on addressing the social determinants of health and acting through proportionate universalism (i.e., making services universally available but directed towards disadvantaged populations) [70]. This review and framework do not point towards specific health issues or determinants to prioritise in addressing health inequalities, which has already been examined in the literature, rather it has assessed the principles of efficacy which appear to carry over across many levels, types, and domains of action to reduce inequalities.

### 4.4 Strengths and limitations

This rapid review assessed evidence from a broad range of umbrella reviews, systematic reviews, primary studies, and grey literature which covered a variety of domains related to health inequalities. This methodology enabled high-level analysis of principles which might impact levelling up health efforts. For the first time, this report brings together a set of practical principles for acting to level up health based on an expansive evidence base. Furthermore, the framework is applicable at many levels from national to local governments and across sectors from non-profit organisations to community institutions. As the principles are broad in scope, they can be applied to any effort to reduce health inequalities and are not constrained by any one domain. The framework is also highly relevant to policy making. Drawing upon an expert panel during the design and interpretation of the research increases the quality.

This review was limited by a general gap in data availability and evaluation of how interventions impact health inequalities. As a rapid review with many levels of included evidence, there was likely a varying degree of quality research included which may have impacted assessment of the evidence base overall. Additionally, some literature may have been missed due to the nature of rapid methodology used. While no formal quality assessment of the included studies was undertaken it was noted in the literature that: many umbrella and systematic reviews did not differentiate results by level of disadvantage; there was a lacking consensus on how to define and measure disadvantage resulting in an incomplete picture of health inequality and leaving unaddressed the nuances of varied health inequality pathways and how intersecting vulnerabilities may be compounded; and there were many shorter-term evaluations reviewed which might not have captured the true impact of interventions. However, the purpose of the review was not to identify and appraise discrete interventions, but rather to identify general patterns in the data to guide policy making. To this end, these limitations are likely to have less of an impact compared to a traditional systematic review.

### 4.5 Research and policy recommendations

The literature on inequalities remains imbalanced on describing the problem of inequalities rather than finding solutions. More detailed research is needed on specific programme and policy impacts and via what mechanisms they reduce inequalities. Future research should collect more robust data assessing how intervention impact is distributed across different levels and types of disadvantage. Further research is needed to examine the extent to which the UK levelling up programme aligns with these guiding principles.

Policy-makers should focus on long-term, collaborative and cross-government strategies; the ambition to level up health will not be achieved in one electoral cycle. Efforts to address health inequalities across and within countries will require action from different actors and sectors to address the multiple wider determinants of health. National and local policies to level up should be informed and checked against these evidence-based levelling up for health principles, for example within the health inequalities impact assessment process [71]. The government should prioritise those interventions, such as widespread fluoridation of water and pollution reduction, which create healthier conditions for all. Local community engagement is fundamental. This requires building long-term relationships and trust with communities, and ensuring representation reflects the diversity of each community. Bespoke initiatives for communities facing specific issues are needed alongside universal initiatives ensuring that resources, such as funding, staff time or estates, are allocated proportionate to need is imperative to levelling up.

## 5 Conclusions

The pandemic has exposed and exacerbated health inequalities. It is paramount that action is taken to reduce health inequalities, closing the gap between those who experience good and poor health while also improving health for all. Here we present a framework of guiding principles based on a high-level rapid review of the evidence to inform levelling up health. These five principles are 1) **healthy-by-default and easy to use initiatives**; 2) **long-term, multi-sector action**; 3) **locally designed focus**; 4) **targeting disadvantaged communities**; and 5) **matching of resources to need.** These principles can and should be applied to the efforts of recovering from and rebuilding after the pandemic and more research is needed to assess the extent to which health inequalities actions align to this framework.

## Funding

This research was externally commissioned by Public Health England.

## Declaration of competing interest

The authors declare that they have no known competing financial interests or personal relationships that could have appeared to influence the work reported in this paper.

## Figures and Tables

**Table:** 

Level 1	Level 2	Level 3
**Wider Economic**	• Labour market	• Wage levels • Job quality • Job availability • Unemployment
	• Welfare system	• Poverty strategies • Active labour market policies
**Social**	• Opportunity structures	• Voluntary sector provision • Education and childcare • Food availability • Health care services • Housing quality/cost
	• Collective social functioning	• Community control • Social capital and assets • Place-based stigma • Culture • Crime and safety
**Wider Policy and Politics**	• Public health regulation • Health care system • Social policies	
**Local Economic**	• Economic policies • Investment	
**Wider Environmental**	• Climate crisis	
**Local Physical**	• Housing • Workplace • Air pollution • Contaminated land • Green space • Infrastructure & transport • Built environment	
**Health related practices**	• Smoking • Drugs and alcohol • Physical activity • Diet • Gambling	

## Appendix 2 MEDLINE Search strategy

Ovid MEDLINE(R) and Epub Ahead of Print, In-Process, In-Data-Review & Other Non-Indexed Citations, Daily and Versions(R) < 1946 to July 27, 2021>

1 ((overview$ or review or synthesis or summary or Cochrane or analysis) and (reviews or meta-analyses or articles or umbrella)).ti. or "umbrella review".ab. or (meta-review or metareview).ti,ab. 6193
2 Residence Characteristics/or Environment design/or exp Marital status/or neighbo?rhood*.ti,ab. or residential environment*.ti,ab. or rural*.ti,ab. or inner?city.ti,ab. or housing instability.ti,ab. or housing insecurity.ti,ab. or housing strain.ti,ab. or housing security.ti,ab. or mortgage problems.ti,ab. or foreclosure.ti,ab. or eviction*.ti,ab. or housing loss.ti,ab. or home repossession*.ti,ab. or home ownership.ti,ab. or (repossess* adj3 hous*).ti,ab. or (repossess* adj3 propert*).ti,ab. or mortgage delinquency.ti,ab. or mortgage arrears.ti,ab. or mortgage debt*.ti,ab. or overcrowding.ti,ab. or (living adj1 (outside or inside or near* or adjacent)).ti,ab. or (household adj2 size).ti,ab. or (marital status or marriage status).ti,ab. or (widow* or cohabit* or divorce* or single parent* or live* alone).ti,ab. 288538
3 Cultural Deprivation/or Acculturation/or Culture/or Cross-Cultural Comparison/or Cultural Characteristics/or Cultural Diversity/or Language/or "Transients and Migrants"/or exp "Emigrants and Immigrants"/or Minority groups/or Minority health/or Prejudice/or Racism/or Xenophobia/or Social Discrimination/or exp Race Relations/or exp Ethnic Groups/or exp Continental Population Groups/or Refugees/or minorit*.ti,ab. or migration background.ti,ab. or racial.ti,ab. or racism.ti,ab. or ethnology.ti,ab. or race.ti,ab. or ethnic*.ti,ab. or non?English.ti,ab. or language other than.ti,ab. or latino*.ti,ab. or latina*.ti,ab. or hispanic*.ti,ab. or whites.ti,ab. or caucasian*.ti,ab. or non?white.ti,ab. or Torres Strait Islander.ti,ab. or aboriginal.ti,ab. or native american.ti,ab. or inuit.ti,ab. or eskimo.ti,ab. or first nation*.ti,ab. or indigenous.ti,ab. or english as a second language.ti,ab. or foreign language.ti,ab. 755621
4 Occupations/or Unemployment/or occupations.ti,ab. or unemployment.ti,ab. 47802
5 exp Educational status/or Education/or Schooling.ti,ab. or educational status.ti,ab. or (education* adj2 level?).ti,ab. or ((higher or better or worse or less) adj educated).ti,ab. or ((higher or better or worse or less) adj level? of education).ti,ab. 131123
6 Religion/or religi*.ti,ab. 47654
7 Social determinants of Health/or Psychosocial Deprivation/or Sociological Factors/or Working Poor/or Hierarchy, Social/or disparit*.ti,ab. or inequalit*.ti,ab. or inequit*.ti,ab. or equity.ti,ab. or deprivation.ti,ab. or gini.ti,ab. or concentration index.ti,ab. or Socioeconomic Factors/or Social Welfare/or exp Social Class/or exp Poverty/or Income/or Social class*.ti,ab. or social determinants.ti,ab. or social status.ti,ab. or social position.ti,ab. or social background.ti,ab. or social circumstance*.ti,ab. or socio-economic.ti,ab. or socioeconomic.ti,ab. or sociodemographic.ti,ab. or socio-demographic.ti,ab. or SES.ti,ab. or disadvantaged.ti,ab. or impoverished.ti,ab. or impoverished.ti,ab. or economic level.ti,ab. or assets index.ti,ab. or income*.ti,ab. 642636
8 Social Stigma/or social capital/or Social Control, Informal/or exp Social Support/or exp Social Environment/or Trust/or Social conditions/or Social isolation/or Social marginalization/or Anomie/or social participation/or social exclusion.ti,ab. or (social adj (capital or cohes* or organis* or organiz*)).ti,ab. or (community adj3 (cohes* or participa*)).ti,ab. or ((neighbourhood or neighbourhood) adj cohes*).ti,ab. or social relationships.ti,ab. or social network*.ti,ab. or collective efficacy.ti,ab. or civil society.ti,ab. or informal social control.ti,ab. or neighbo*rhood disorder.ti,ab. or social disorgani?ation.ti,ab. or anomie.ti,ab. or social support.ti,ab. or social participation.ti,ab. or trust.ti,ab. or emotional support.ti,ab. or psychosocial support.ti,ab. or community capital.ti,ab. or neighbo*rhood cohesion.ti,ab. or social influence.ti,ab. or (soci*context* or soci*-context*).ti,ab. 275159
9 Health Status Disparities/or Health Services Accessibility/or Health Equity/or health*care disparit*.ti,ab. or health care disparit*.ti,ab. or health status disparit*.ti,ab. or health disparit*.ti,ab. or health inequalit*.ti,ab. or health inequit*.ti,ab. or medically underserved.ti,ab. or (digital adj3 (exclud* or exclud* or access* or divide)).ti,ab. or exp digital divide/113520
10 ((Gypsy* or gypsies or gipsy* or gipsies) not (moth or moths)).ti,ab. 1838
11 (Roma or romas or romany or romani or romanis or romanies or romanian).ti,ab. 4581
12 (circus* or (bargee* or canal boat* or barge* or boat-dwell*) or (pavee* or minceir* or lucht* or luchd* or itinerant*) or (travel?er* and (communit* or family or families or irish or ireland* or eire or wales or welsh or scottish or scotland* or highland* or norwegian* or norway* or newage or new-age or itinerant* or minorit* or ethnic* or halting site* or caravan*)) or (travel?ing adj5 (communit* or family or families or irish or ireland* or eire or wales or welsh or scottish or scotland* or highland* or norwegian* or norway* or newage or new-age or itinerant* or minorit* or ethnic* or site* or caravan*))).ti,ab. 3921
13 (("population level" or "population based" or "population orientated" or "population oriented" or "community level" or "community based" or "community orientated" or "community oriented") adj8 (intervention$ or prevention or policy or policies or program$ or project$)).tw. 26088
14 (health adj8 (intervention$ or prevention or policy or policies or program$ or project$)).tw. 254766
15 ((health care system or social care) and (funding or financial or pooling or insurance or insured or provider or provision or tax or taxation or budget or pay or commission or purchasing or purchaser or market or marketization or privatization or marketization or privatization) and (equity or socioeconomic or socio-economic or equality or ses or SES or deprivation or deprived or education or income or poverty or poor or unemployed or social class or occupation)).mp. [mp = title, abstract, original title, name of substance word, subject heading word, floating sub-heading word, keyword heading word, organism supplementary concept word, protocol supplementary concept word, rare disease supplementary concept word, unique identifier, synonyms] 3658
16 (health or wellbeing or well-being or mortality or "life expectancy" or morbidity or disease or incidence or prevalence or illness or death).mp. 9292151
17 Health Status/or Health Status Disparities/or Morbidity/or Mortality/or Incidence/or Prevalence/724871
18 16 or 17 9292151
19 (Econom* factor or Econom* determinant or Econom* growth or econom* development or macroeconom* or macro econom* or economic integration or economic globalization or economic globalisation or Transitional Econom*).tw. 13347
20 (market structure or market design or pricing or freemarket or free market or market* Competition or Monopoly or Oligopoly or financial markets or trade policy or international trade or international factor movements or international business or Remittances or international finance or financial transactions tax or taxation or tax evasion or evasion of tax or tax avoid* or marketing or advertising or antitrust or trade or business cycle or business fluctuation or remittances or externalities).tw. 92507
21 (economic institutions or multinational firm* or central bank* or Banking or banks or depository institutions or business economics or IMF or WTO).tw. 17549
22 (money supply or credit supply or supply of credit or interest rate* or rate* of interest or financial polic* or financialization or financialization or financial services or financial institutions or financial cris* or corporate governance or corporate finance or fiscal or lending or debt or micro finance or mortgage* or monet* or inflation or deflation or structural adjustment or trade deficit or budget* deficit or investment or economic recession or currenc* or price level or monetary or international lending or foreign aid or national budget or national deficit or national debt or capital).tw. 109232
23 (Scope of Government or Social Security or underground economy or welfare programs or entrepreneurship or non profit or nonprofit or informal econom* or land ownership or land reform or shadow econom* or informal econom* or alternative econom or informal sector or urban econom* or regional econom* or rural econom* or Nationalization or Nationalization or Privatization or Privatization or Government Expenditure* or Size of Government or social enterprise* or public enterprise* or private enterprise* or Land Ownership or ownership of land or Land Tenure or Land Reform or public investment or Property rights or Open Econom* or subsid* or public good or cooperative enterprises or Welfare state).tw. 50381
24 (Firm Objectives or objectives of the firm or objectives of firms or organization of firms or organization of the firm or organisation of firms or organisation of the firm or Firm Organization or Firm Organisation or Firm Behavior or Firm Behaviour or behaviour of firms or behaviour of the firm or behavior of firms or behavior of the firm or retirement or compensation package* or trade union or labor managed firm* or labour managed firm* or Worker* Rights or rights of workers or employee managed firm or employee owned Firms or firm performance or wage* or Human Capital or income* or employment or unemployment or enterprises or entrepreneur* or labor demand or labour demand or demand for labor or demand for labour or labor economics or labour economics or labor supply or labour supply or supply of labor or supply of labour or labor discrimination or labour discrimination or cost of labor or cost of labour or labor cost* or labour cost* or labor mobility or labour mobility or labor market or labour market or labor standards or labour standards or labor force size or labour force size or size of the labor force or size of the labour force or labour force structure or labour force structure or structure of the labor force or structure of the labour force or labor management relations or labour management relations).tw. 233325
25 (resource distribution or distribution of resources or economic justice or externalit* or Gross Domestic Product or gross national income or industrialization or industrialization or industrial structure or industrial policy or industrial ecology or poverty or wealth or economic inequalit* or production of goods or production of services or means of production or consumption of goods or consumption of services or pattern* of consumption or productivity or manufacturing or startups or social status).tw. 177249
26 (socialist or socialism or Public Economics or Welfare Economics or environmental economics or ecological economics or Marx* or Keynes* or Neoclassic* or capitalism or capitalist or neoliber* or political economy or economic austerity or (economic recession or degrowth)).tw. 10291
27 Socioeconomic Factors/or Income/or Employment/or Poverty/or Social Class/or Economics/302716
28 19 or 20 or 21 or 22 or 23 or 24 or 25 or 26 or 27 833237
29 18 and 28 482599
30 Income/or Social Welfare/or Social Security/or Financial Support/or Public Assistance/or Financing, Government/72536
31 (welfare or poverty or low income or standard of living or minimum wage* or minimum salar* or debt relief or income support or income maintenance or cash transfer* or deprivation or social exclusion or social inclusion or social protection or public assistance or social assistance or disability insurance or disab* NEAR insurance or social securit* or safety NEAR net* or pension* or old age or retirement benefit or social assistance or social insurance or micro NEAR insurance or disabilit* NEAR grant* or disabilit* NEAR benefit* or social NEAR health NEAR protection* or sickness or long term ill* or work NEAR injur* or employment NEAR injur* or work compensation or health insurance or child benefit or lone parent or single parent or parental leave or maternity leave or paternity leave or family benefit or family polic* or food stamp* or food subsid*).tw. 289187
32 (unemployment or workless* or jobless* or income support or jobseekers allowance or employment status or full employment or labour market polic* or labor market polic* or vocational train* or vocational education or vocational rehabilitation or economic activity or welfare).tw. 64336
33 30 or 31 or 32 364389
34 (inequality or inequalities or equality or inequity or inequities or equity or disparity or disparities or gap or gaps or gradient or gradients or unequal or disadvantage* or variation* or socioeconomic or socio-economic or SES or disab* or poverty or deprivation or deprived or social determinants or underserved population* or minorit* or immigrant* or racial or ethnic*).tw. 1991559
35 33 and 34 155996
36 ((health or death or mortality or disease or ill* or morbidity or injur* or accident* or casualt*) and (traffic calming or traffic-calming or traffic or traffic speed or speed limit or speed reduction or speed camera or speed hump or road hump or roundabout or road design or road modification or road environment or street environment or 20 mph)).mp. [mp = title, abstract, original title, name of substance word, subject heading word, floating sub-heading word, keyword heading word, organism supplementary concept word, protocol supplementary concept word, rare disease supplementary concept word, unique identifier, synonyms] 63928
37 Gambling/or gambl*.tw. 11067
38 (("physical* activ*" or sport* or walking or exercise or lifestyle or "life style" or "physical fitness" or "motor activi*") and ("low SES" or "low* socio*" or "low* income" or disadvantaged or inequal* or disparity or deprived or underserved or "low* educat*" or poverty or "social class" or equity)).mp. [mp = title, abstract, original title, name of substance word, subject heading word, floating sub-heading word, keyword heading word, organism supplementary concept word, protocol supplementary concept word, rare disease supplementary concept word, unique identifier, synonyms] 18572
39 exp Obesity/or exp Body Weight/or exp Body Weight Changes/or exp Weight Gain/or exp Weight Loss/or (obese or obesity).tw. or overweight.tw. or weight.tw. or diet$.tw. or nutrition$.tw. or (physical$ adj activ$).tw. or exercise$.tw. or lifestyle$.tw. or (bmi$ or (body adj mass ind$)).tw. or (waist adj6 circumference$).tw. or ((weight adj2 (control or reduction) adj2 (advice or counsel$ or program$ or intervention?)) or (weight adj manag$)).tw. or ((overweight or obese or obesity) adj4 (Advice or counsel$ or intervention? or program$)).tw. 2320155
40 exp Smoking/or exp Smoking Cessation/or nicotine.tw. or cigarette$.tw. or (nicotine replacement therapy or NRT).tw. or smoking cessation.tw. or smok$.tw. or exp "Tobacco Use Cessation"/or exp Smoking Cessation/or (smoking cessation or (quit$ adj2 smok$)).tw. or ((reduce or reducing) adj3 ('tobacco use' or cigarette? or smoking or addiction)).tw. 367223
41 alcohol.mp. or exp Alcohols/or exp Alcohol Drinking/or exp Alcoholism/or exp Drinking Behavior/or drink$.tw. or beer.tw. or wine.tw. or ethanol.tw. or drunk.tw. or (addict$ or (alcohol adj2 (abus$ or misus$))).tw. or alcohol$.tw. or drunk$.tw. or intoxicat$.tw. 1240595
42 39 or 40 or 41 3617332
43 34 and 42 311355
44 Health Promotion/or health promotion.ti,ab. or health behaviour.ti,ab. or health behavior.ti,ab. or (policy and (social or school or food or public or urban or environmental or fiscal)).ti,ab. or urban planning.ti,ab. or city planning.ti,ab. or built environment.ti,ab. or social environment.ti,ab. or physical environment.ti,ab. or cultural environment.ti,ab. or urban environment.ti,ab. or school environment.ti,ab. or neighbourhood.ti,ab. or community.ti,ab. or societal.ti,ab. or social interventions.ti,ab. or community interventions.ti,ab. or obesogenic environment.ti,ab. or individual level.ti,ab. or lifestyle.ti,ab. or individual.ti,ab. or tax$.ti,ab. or subsid$.ti,ab. or price$.ti,ab. or health education.ti,ab. or social marketing.ti,ab. or (diet and (advice or counselling)).ti,ab. or (exercise and (advice or counselling)).ti,ab. or weight management.ti,ab. or cash transfer$.ti,ab. or lifestyle counselling.ti,ab. or behavioural counselling.ti,ab. or behavioral counselling.ti,ab. or exercise on prescription.ti,ab. or exercise.ti,ab. or health trainer$.ti,ab. or school.ti,ab. or workplace.ti,ab. or campaign$.ti,ab. or (access adj1 facilities).ti,ab. or green space.ti,ab. or walk?ability.ti,ab. or food label$.ti,ab. or food advert$.ti,ab. 2298386
45 (BMI or Body Mass Index).ti,ab. or Body Weight/or obesity.ti,ab. or obese.ti,ab. or overweight.ti,ab. or weight gain.ti,ab. or weight loss.ti,ab. or exp OBESITY/or Body fat.ti,ab. or Fat mass.ti,ab. or Weight control$.ti,ab. or Weight maintain$.ti,ab. or Adipos$.ti,ab. or Adipose tissue.ti,ab. or Skinfold thickness.ti,ab. or Waist circumference.ti,ab. or Waist hip ratio.ti,ab. or WHR.ti,ab. 867665
46 ″Body Weights and Measures"/6717
47 45 or 46 871478
48 44 or 47 3023418
49 34 and 48 430648
50 ((communit* or citizen* or public* or minorit* ethnic* or stakeholder* or population* or neighbourhood or neighbourhood) adj5 (engag* or develop* or empower* or involv* or participat* or collaborat* or consult* or partner* or forum* or panel* or jury or champion*)).mp. [mp = title, abstract, original title, name of substance word, subject heading word, floating sub-heading word, keyword heading word, organism supplementary concept word, protocol supplementary concept word, rare disease supplementary concept word, unique identifier, synonyms] 176489
51 (austerity or welfare benefit or welfare reform or Social assistance).mp. or exp Social Security/or Social security.mp. or Income benefits.mp. or Income support.mp. or Income supplement.mp. or Income maintenance.mp. or Pensions/or Conditional cash.mp. or Cash assistance.mp. or Unemployment benefit.mp. or Child Benefit.mp. or Tax credit.mp. or Family benefit.mp. or Family support.mp. or Conditionality.mp. or Poverty reduction.mp. or Housing benefit.mp. or Anti-poverty.mp. or Family allowance.mp. or Entitlement.mp. or Generosity.mp. or Disability benefit.mp. 30391
52 2 or 3 or 4 or 5 or 6 or 7 or 8 or 9 or 10 or 11 or 12 or 13 or 14 or 15 or 29 or 35 or 37 or 38 or 43 or 50 or 51 2395886
53 1 and 52 1206
54 limit 53 to yr = "2007 -Current" 1145

## References

[1] World Health Organization https://www.who.int/news-room/facts-in-pictures/detail/health-inequities-and-their-causes.

[2] (2020). https://www.gov.uk/government/publications/covid-19-review-of-disparities-in-risks-and-outcomes.

[3] Bambra C., Riordan R., Ford J., Matthews F. (2020). The COVID-19 pandemic and health inequalities. J. Epidemiol. Community Health.

[4] Yaya S., Yeboah H., Charles C.H., Otu A., Labonte R. (2020). Ethnic and racial disparities in COVID-19-related deaths: counting the trees, hiding the forest. BMJ Glob Heal.

[5] (2020). https://www.ons.gov.uk/peoplepopulationandcommunity/birthsdeathsandmarriages/deaths/bulletins/deathsinvolvingcovid19bylocalareasanddeprivation/deathsoccurringbetween1marchand17april.

[6] Bibby J., Everest G., Abbs I. (2020). https://www.health.org.uk/publications/long-reads/will-covid-19-be-a-watershed-moment-for-health-inequalities.

[7] (2021). Unemployment - Ethnicity Facts and Figures.

[8] https://www.gov.uk/government/news/prime-minister-hails-levelling-up-in-action-as-government-unveils-raft-of-new-policies.

[9] Communities, Department for Levelling Up Housing (2022).

[10] Thornton R.L.J., Glover C.M., Cené C.W., Glik D.C., Henderson J.A., Williams D.R. (2016). Evaluating strategies for reducing health disparities by addressing the social determinants of health. Health Aff..

[11] Pons-Vigués M., Diez È., Morrison J., Salas-Nicás S., Hoffmann R., Burstrom B. (2014). Social and health policies or interventions to tackle health inequalities in European cities: a scoping review. BMC Publ. Health.

[12] Bambra C., Joyce K.E., Bellis M.A., Greatley A., Greengross S., Hughes S. (2010). Reducing health inequalities in priority public health conditions: using rapid review to develop proposals for evidence-based policy. J. Public Health.

[13] Bambra C., Gibson M., Sowden A., Wright K., Whitehead M., Petticrew M. (2010). Tackling the wider social determinants of health and health inequalities: evidence from systematic reviews. J. Epidemiol. Community Health.

[14] Bambra C. (2016).

[15] Gibson M., Petticrew M., Bambra C., Sowden A.J., Wright K.E., Whitehead M. (2011).

[16] Cairns J., Warren J., Garthwaite K., Greig G., Bambra C. (2015). Go slow: an umbrella review of the effects of 20 mph zones and lints on health and health inequalities. J. Public Health.

[17] Thomson K., Hillier-Brown F., Todd A., McNamara C., Huijts T., Bambra C. (2018). The effects of public health policies on health inequalities in high-income countries: an umbrella review. BMC Publ. Health.

[18] Naik Y., Baker P., Ismail S.A., Tillmann T., Bash K., Quantz D. (2019). Going upstream – an umbrella review of the macroeconomic determinants of health and health inequalities. BMC Publ. Health.

[19] McCartney G., Hearty W., Arnot J., Popham F., Cumbers A., McMaster R. (2019). Impact of political economy on population health: a systematic review of reviews. Am. J. Publ. Health.

[20] Carey G., Crammond B., Malbon E. (2019). Personalisation schemes in social care and inequality: review of the evidence and early theorising. Int. J. Equity Health.

[21] Macintyre A.K., Torrens C., Campbell P., Maxwell M., Pollock A., Biggs H. (2020). Socioeconomic inequalities and the equity impact of population-level interventions for adolescent health: an overview of systematic reviews. Publ. Health.

[22] Garzón-Orjuela N., Samacá-Samacá D.F., Luque Angulo S.C., Mendes Abdala C.V., Reveiz L., Eslava-Schmalbach J. (2020). An overview of reviews on strategies to reduce health inequalities. Int. J. Equity Health.

[23] Welch V., Petkovic J., Pardo Pardo J., Rader T., Tugwell P. (2016). Interactive social media interventions to promote health equity: an overview of reviews. Heal Promot Chronic Dis Prev Canada.

[24] Haby M.M., Chapman E., Clark R., Galvão L.A.C. (2016). Agriculture, food, and nutrition interventions that facilitate sustainable food production and impact health: an overview of systematic reviews. Rev. Panam. Salud Públic.

[25] Cauchi D., Glonti K., Petticrew M., Knai C. (2016). Environmental components of childhood obesity prevention interventions: an overview of systematic reviews. Obes. Rev..

[26] Anderson P., Jané-Llopis E., Hasan O.S.M., Rehm J. (2018). City-based action to reduce harmful alcohol use: review of reviews. F1000Research..

[27] Bird E.L., Ige J.O., Pilkington P., Pinto A., Petrokofsky C., Burgess-Allen J. (2018). Built and natural environment planning principles for promoting health: an umbrella review. BMC Publ. Health.

[28] Craike M., Wiesner G., Hilland T.A., Bengoechea E.G. (2018). Interventions to improve physical activity among socioeconomically disadvantaged groups: an umbrella review. Int. J. Behav. Nutr. Phys. Activ..

[29] Pierron A., Fond-Harmant L., Laurent A. (2018). Alla F cois. Supporting parenting to address social inequalities in health: a synthesis of systematic reviews. BMC Publ. Health.

[30] Thomson K., Hillier-Brown F., Walton N., Bilaj M., Bambra C., Todd A. (2019). The effects of community pharmacy-delivered public health interventions on population health and health inequalities: a review of reviews. Prev. Med..

[31] Petticrew M., Bambra C., Gibson M., Sowden A., Whitehead M., Wright K. (2008).

[32] (2014). Nine Local Actions to Reduce Health Inequalities. London.

[33] Gledhill R. (2018).

[34] (2020). Social Determinants of Health and the Role of Local Government.

[35] (2020). Public Health Transformation Seven Years on: Prevention in Neighbourhood, Place and System.

[36] Honeyman M., Maguire D., Evans H., Davies A. (2020).

[37] Naik Y., Abbs I., Elwell-Sutton T., Bibby J., Spencelayh E., Shafique A. (2020).

[38] Marmot M., Allen J., Boyce T., Goldblatt P., Morrison J., Michael Marmot B. (2020).

[39] Council C.C. (2020).

[40] Marmot M., Allen J., Boyce T., Goldblatt P., Morrison J. (2021).

[41] Qureshi A., Longlands S. (2021).

[42] (2015). Tackling Inequalities in the Early Years: Key Messages from 10 Years of the Growing up in Scotland Study. Edinburgh.

[43] Allen M., Allen J. (2015).

[44] Health inequalities (2015).

[45] Baker A., Bentley C., Carr D., Connolly A.M., Heasman M., Johnson C. (2017).

[46] Pearce-Smith N. (2018).

[47] Tran A. (2018).

[48] De Brún C. (2018).

[49] Hyland A. (2018).

[50] Eyles H., Ni Mhurchu C., Nghiem N., Blakely T. (2012). Food pricing strategies, population diets, and non-communicable disease: a systematic review of simulation studies. PLoS Med..

[51] Brown T., Platt S.A.A. (2014). Equity impact of European individual-level smoking cessation interventions to reduce smoking in adults: a systematic review. Eur. J. Publ. Health.

[52] Crocker-Buque T., Edelstein M., Mounier-Jack S. (2017). Interventions to reduce inequalities in vaccine uptake in children and adolescents aged <19 years: a systematic review. J. Epidemiol. Community Health.

[53] Simpson J., Albani V., Bell Z., Bambra C., Brown H. (2021). Effects of social security policy reforms on mental health and inequalities: a systematic review of observational studies in high-income countries. Soc. Sci. Med..

[54] Beauchamp A., Backholer K.D., Peeters M.A. (2014). The effect of obesity prevention interventions according to socioeconomic position: a systematic review. Obes. Rev..

[55] Durand M.-A., Carpenter L., Dolan H., Bravo P., Mann M., Bunn F. (2014). Do interventions designed to support shared decision-making reduce health inequalities? A systematic review and meta-analysis. PLo∼S One.

[56] Cairns J.-M., Bambra C., Hillier-Brown F.C., Moore H.J., Summerbell C.D. (2015). Weighing up the evidence: a systematic review of the effectiveness of workplace interventions to tackle socio-economic inequalities in obesity. J. Public Health.

[57] Brown T., Platt S., Amos A. (2014). Equity impact of interventions and policies to reduce smoking in youth: systematic review. Tobac. Control.

[58] Hillier-Brown F.C., Bambra C., Cairns J.-M., Kasim A., Moore H.J., Summerbell C.D. (2014). A systematic review of the effectiveness of individual, community and societal level interventions at reducing socioeconomic inequalities in obesity amongst children. BMC Publ. Health.

[59] Harris J.S.J.C.L. (2015). https://www.ncbi.nlm.nih.gov/books/NBK274412/.

[60] McGill R., Anwar E., Orton L., Bromley H., Lloyd-Williams F., O'Flaherty M. (2015). Are interventions to promote healthy eating equally effective for all? Systematic review of socioeconomic inequalities in impact. BMC Publ. Health.

[61] Moore G.F., Littlecott H.J., Turley R., Waters E., Murphy S. (2015). Socioeconomic gradients in the effects of universal school-based health behaviour interventions: a systematic review of intervention studies. BMC Publ. Health.

[62] Barr B., Bambra C., Whitehead M., Duncan W.H. (2014). The impact of NHS resource allocation policy on health inequalities in England 2001-11: longitudinal ecological study. BMJ.

[63] Barr B., Higgerson J., Whitehead M. (2017). Investigating the impact of the English health inequalities strategy: time trend analysis. BMJ.

[64] Griffin S., Love-Koh J., Pennington B., Owen L. (2019). Evaluation of intervention impact on health inequality for resource allocation. Med. Decis. Making.

[65] Øversveen E., Rydland H.T., Bambra C., Eikemo T.A. (2017). Rethinking the relationship between socio-economic status and health: making the case for sociological theory in health inequality research. Scand. J. Publ. Health.

[66] Lundberg O. (2020). Next steps in the development of the social determinants of health approach: the need for a new narrative. Scand. J. Publ. Health.

[67] Williams O., Fullager S. (2018). Lifestyle drift and the phenomenon of “citizen shift” in contemporary UK health policy. Sociol. Health Illness.

[68] J. P, M. W, Hunter D.J. (2010). Injustice is killing people on a large scale—but what is to be done about it?. J. Public Health.

[69] (2019). Place-based Approaches for Reducing Health Inequalities: Foreword and Executive Summary - GOV.

[70] chair) Marmot M (, Fair Society Health Lives (2010).

[71] Tyler I., Pauly B., Wang J., Patterson T., Bourgeault I., Manson H. (2019). Evidence use in equity focused health impact assessment: a realist evaluation. BMC Publ. Health.

[72] Whitehead M., Bambra C., Barr B., Bowles J., Caulfield R., Doran T. (2014). Due North: report of the inquiry on health equity for the North university of liverpool and centre for local economic strategies, liverpool and manchester [internet]. http://www.cles.org.uk/publications/due-north-report-of-the-inquiry-on-health-equity-for-the-north/.

[73] (2021). Place-based Approaches for Reducing Health Inequalities: Main Report.

